# Radiotherapy combined with immunotherapy achieves clinical complete response in elderly metastatic penile cancer: a case report

**DOI:** 10.3389/fimmu.2026.1783437

**Published:** 2026-03-09

**Authors:** Guizhen Huang, Yanghui Li, Su Li, Long Gong, Hongtao Chen, Xianming Li, Guixiang Liao

**Affiliations:** 1 Guangdong Provincial Clinical Research Center for Geriatrics, Shenzhen Clinical Research Center for Geriatrics, Department of Radiation Oncology, Shenzhen People's Hospital, The Second Clinical Medical College, Jinan University, Shenzhen, China; 2 Department of Radiation Oncology, Medical Institute, Shenzhen University, Shenzhen, China

**Keywords:** immune checkpoint inhibitor, inguinal lymph nodes, penile squamous cell carcinoma, radiotherapy, survival

## Abstract

**Background:**

A combination of local and systemic therapies is used to treat inguinal lymph node metastases post-penile cancer surgery. This multimodal approach may include surgery, chemotherapy, radiotherapy, and immunotherapy.

**Case summary:**

We describe an 89-year-old male with a history of penile cancer status post resection, who presented with multiple metastases to the left inguinal lymph nodes confirmed by pathological biopsy. The patient was treated with a combination of radiotherapy and Pembrolizumab. Following this combined modality therapy, he experienced symptomatic relief and achieved a progression-free survival exceeding 38 months.

**Conclusion:**

This case suggests that concurrent radiotherapy plus Pembrolizumab may achieve durable remission in elderly, chemotherapy-intolerant, metastatic PSCC, but verification in more patients is needed.

## Introduction

Penile squamous cell carcinoma (PSCC) is a rare but impactful malignancy, accounting for approximately 0.4-0.6% of all cancers in men in North America and Europe, with a significantly higher disease burden in parts of South America, Africa, and Asia (1, 2). Major risk factors include human papillomavirus (HPV) infection, phimosis, chronic inflammation, and lack of neonatal circumcision in certain populations (3, 4). The primary management for localized disease is surgical resection, ranging from organ-preserving procedures (e.g., glansectomy, partial penectomy) for early-stage tumors to radical penectomy for advanced local disease (5, 6). The status of the inguinal lymph nodes remains the single most important prognostic factor, guiding the use of therapeutic lymphadenectomy (7). Despite definitive local therapy, a significant proportion of patients, particularly those with high-risk pathological features or nodal involvement, experience disease recurrence. Treatment of recurrent and metastatic PSCC presents a formidable clinical challenge. Historically, options for metastatic disease were limited to palliative platinum-based chemotherapy, which offers modest response rates of approximately 15-32% (8) and poor long-term survival, with a 5-year overall survival (OS) rate of only 5-10% (9, 10). This underscores a critical unmet need for more effective systemic therapies. Recent years have witnessed a paradigm shift in the management of advanced PSCC, fueled by a deeper understanding of its molecular landscape. Notably, a subset of tumors exhibits high PD-L1 expression or genomic features indicative of an inflamed tumor microenvironment. This has paved the way for immune checkpoint inhibitors (ICIs), such as Pembrolizumab and Nivolumab, to emerge as a new standard of care for metastatic or unresectable disease, demonstrating superior survival outcomes compared to chemotherapy in clinical trials (11–13). Consequently, the contemporary management of recurrent PSCC is increasingly characterized by a multimodal approach, integrating surgery, radiotherapy, and novel systemic agents like ICIs, with treatment strategies being tailored through multidisciplinary tumor boards (14, 15).

Radiotherapy, particularly at specific doses and with precise fractionation, can induce a form of cell death in tumor cells called immunogenic cell death. Unlike normal apoptosis, Immunogenic Cell Death is characterized by the surface exposure and release of specific damage-associated molecular patterns (DAMPs), such as calreticulin, ATP, and HMGB1. These molecules act as “danger signals” that effectively turn the dying tumor cell into an *in situ* vaccine (16). They recruit and activate dendritic cells, which then phagocytose tumor antigens, mature, migrate to lymph nodes, and prime tumor-specific naive T cells. This process bridges innate and adaptive immunity, generating a systemic anti-tumor immune response. Importantly, this radiotherapy-induced immune priming often creates a microenvironment that is more receptive to ICIs. ICIs (e.g., anti-PD-1/PD-L1, anti-CTLA-4) work by removing the “brakes” on these activated T cells, preventing tumor-mediated T-cell exhaustion and allowing them to effectively attack cancer cells. Thus, radiotherapy provides the antigenic source and initial immune activation, while immunotherapy sustains and amplifies this response.Moreover, the Abscopal Effect sometimes might be observed after radiothrapy.The “abscopal effect” (from Latin ab - away from, and scopus - target) is a rare but paradigm-shifting phenomenon where localized radiotherapy to one tumor site leads to the regression of metastatic tumors at distant, non-irradiated sites. This systemic effect is fundamentally mediated by the adaptive immune system.radiotherapy-induced tumor antigen release and DAMP signaling lead to the generation of tumor-specific T cells that circulate throughout the body (17). These effector T cells can then infiltrate and attack metastatic lesions that share the same antigens as the primary irradiated tumor. However, this endogenous response is often insufficient or suppressed by the immunosuppressive tumor microenvironment of distant sites. The combination with immunotherapy, especially ICIs, is critical to unleash the full potential of the abscopal effect. Immunotherapy supports the expansion, survival, and functionality of these radiation-primed T cells and counteracts immunosuppressive mechanisms systemically, thereby making the abscopal effect more clinically observable and potent. In essence, radiotherapy initiates a systemic immune response, while immunotherapy removes the barriers to its execution at distant sites (18).In the phase II PERICLES trial (19), investigating the combination of atezolizumab and radiotherapy in advanced penile cancer(N = 32) yielded a 1-year progression-free survial (PFS) of 12.5% and a median OS of 11.3 months.

PSCC is a rare genitourinary malignancy with limited therapeutic options in the advanced setting. Although cisplatin-based combination chemotherapy remains the standard of care for fit patients, a substantial proportion of patients are elderly, present with comorbidities, or have poor performance status, rendering them ineligible for platinum-based regimens. For this vulnerable population, no established standard treatment exists, and prognosis remains dismal. The therapeutic dilemma is further compounded in very elderly patients (≥80 years), who are often underrepresented in clinical trials and for whom data on both efficacy and tolerability of modern systemic therapies are extremely scarce. In this context, innovative, well-tolerated, and effective treatment strategies are urgently needed. Here, we report a case of an 89-year-old, chemotherapy-ineligible patient with metastatic PSCC successfully treated with radiotherapy combined with pembrolizumab, achieving a durable complete response exceeding 38 months.

## Case report

**Figure 1:**
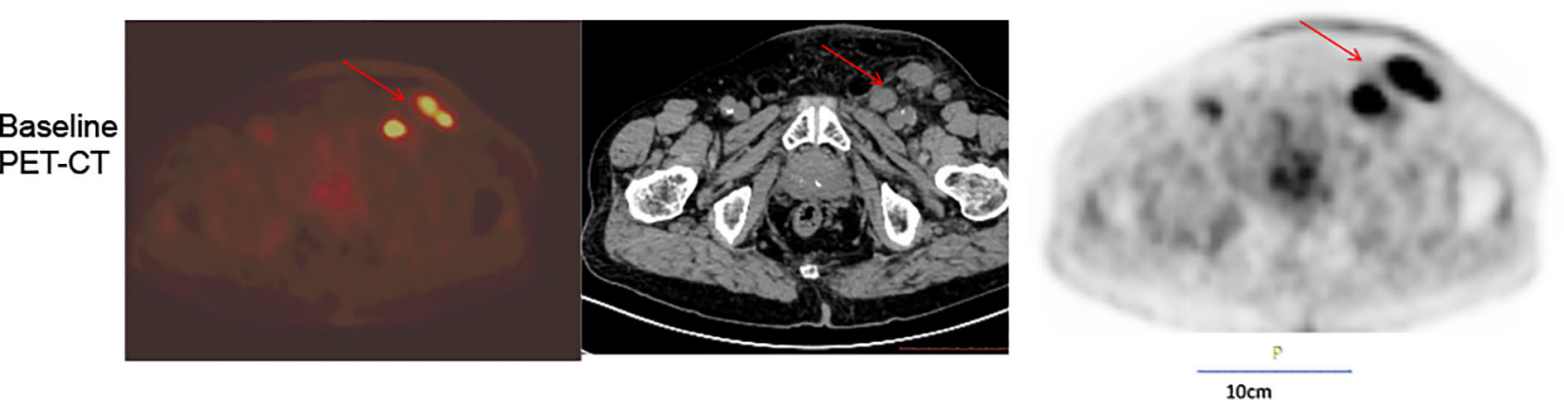
Baseline PET-CT.Multiple enlarged lymph nodes were noted adjacent to the left external iliac vessels in the inguinal region and with increased FDG avidity by PET-CT scan.

**Figure 2:**
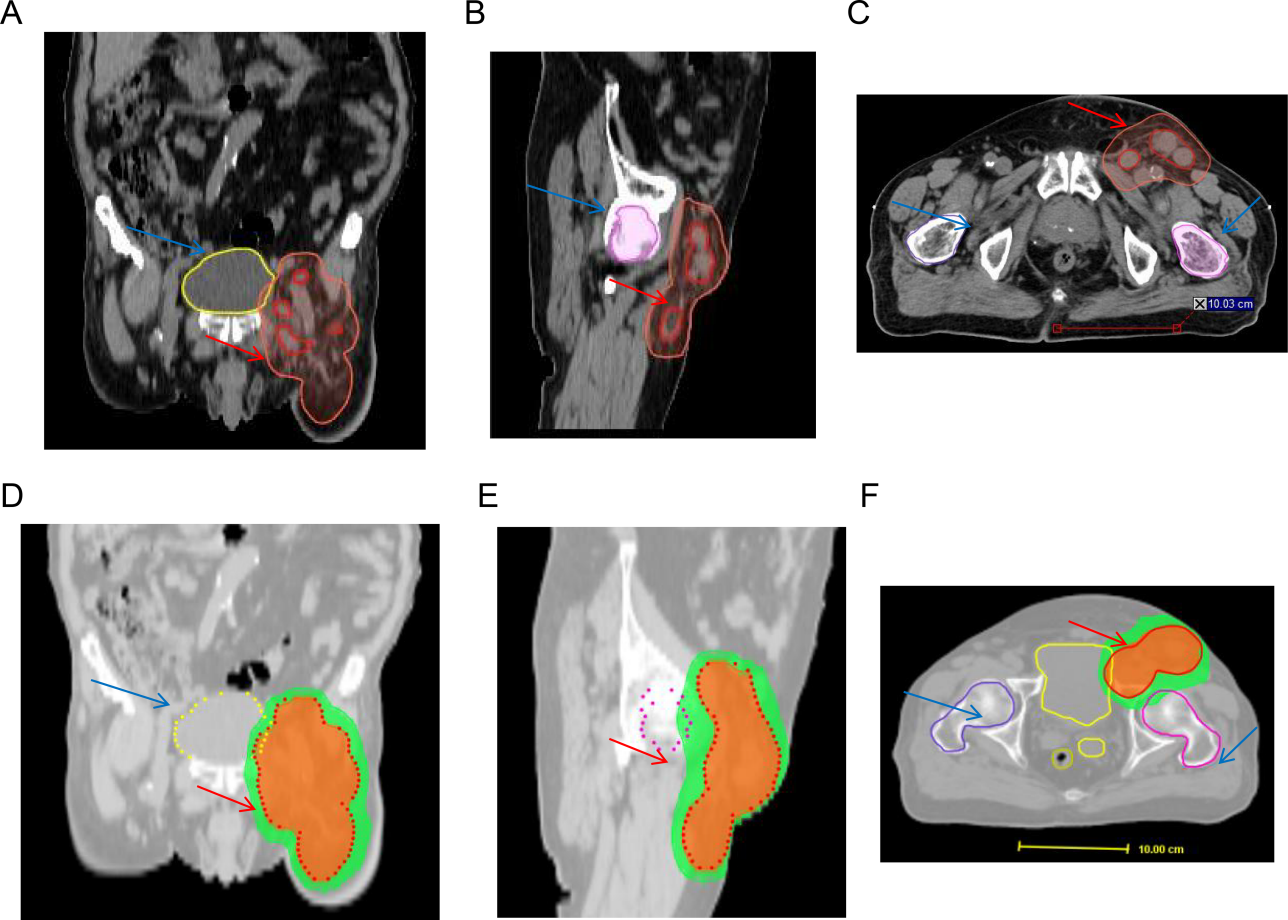
Target volume and dose distribution maps in thecoronal **(A, D)**, sagittal **(B, E)**, and axial planes **(C, F)**. The orange area represents the region receiving a dose coverage of 60 Gy.The green area indicates the region covered by a dose of 45 Gy.The red contour delineates the metastatic inguinal lymph node.The light red contour outlines the Planning Target Volume (PTV).The light blue structure corresponds to the right femoral head, while the left femoral head is depicted in magenta.The yellow contour represents the bladder.

**Figure 3:**
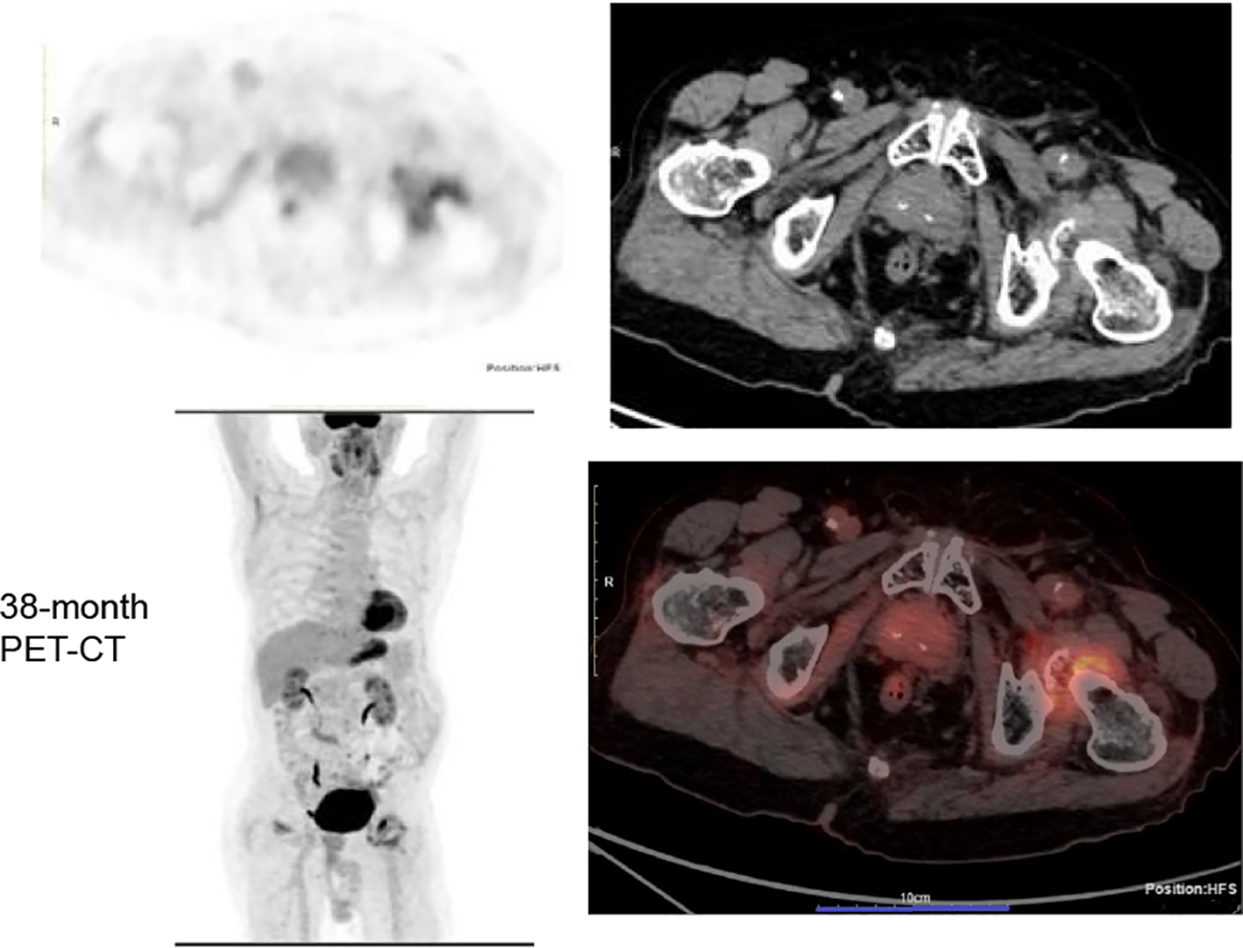
38-month followed-up PET-CT.No evidence of local tumor recurrence at the primary surgical site and no FDG-avid lymph nodes in the left inguinal region by PET/CT scan.

**Figure 4:**
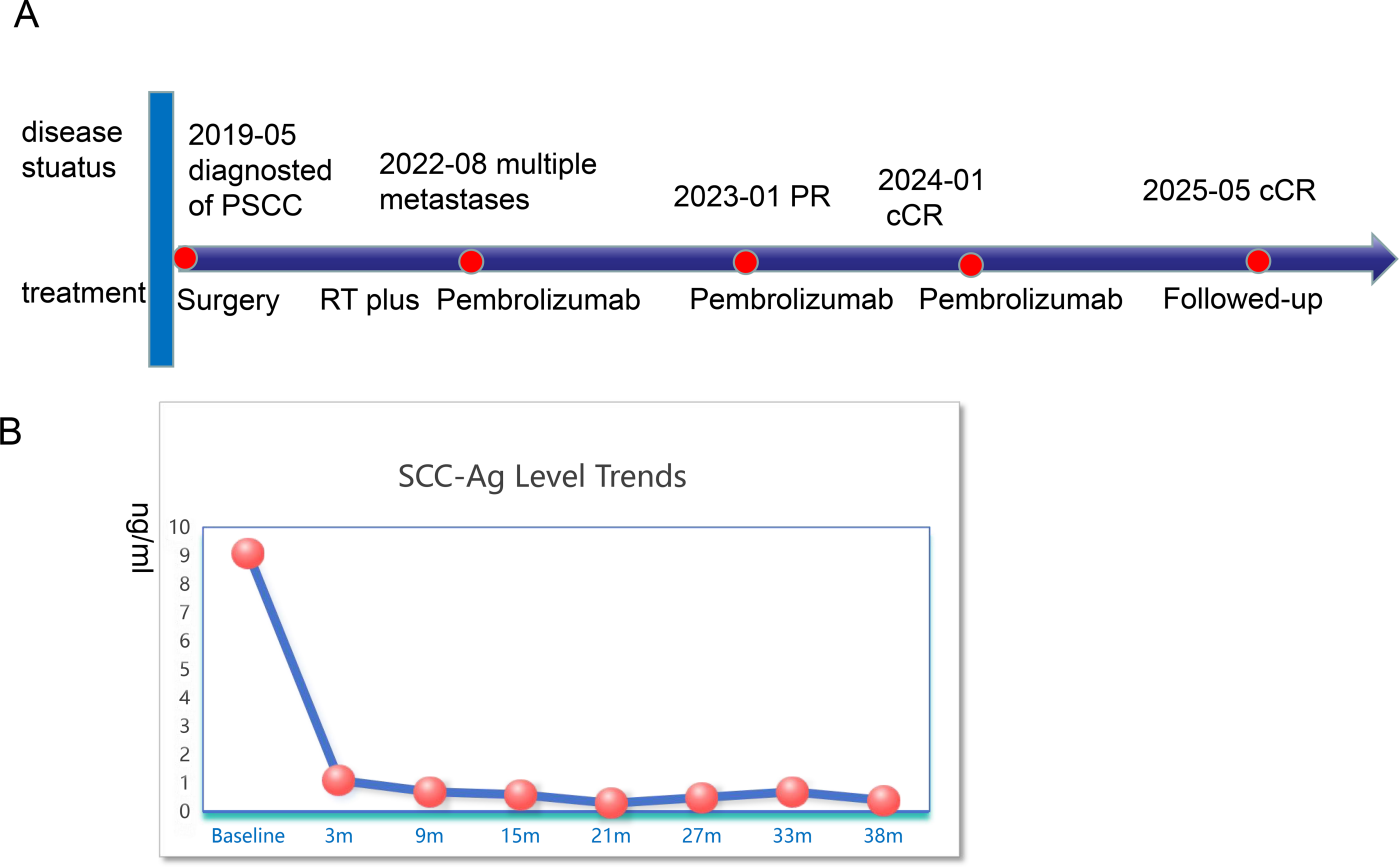
Major clinical event of the patient since diagnosis **(A)** and the SCC-Ag Level Trends **(B)**. cCR, clinical completed response; PR, partial response; PSCC, Penile squamous cell carcinoma; SCC-Ag, squamous cell carcinoma antigen.

This case report was conducted per the CARE Guidelines (20). It was an 89-year-old male, the patient was diagnosed with penis cancer in May 2019 and received Partial Penectomy. The postoperative pathology showed that Tumor size was 4 × 3.5 × 3 cm. It was PSCC, moderately differentiated, negative for lymphovascular and perineural invasion, diagnosed as pT2N0M0. He did not receive any postoperative adjuvant therapy but underwent regular follow-up. In August 2022, he was hospitalized because of edema of the left lower extremity. The ECOG performance score was 1 at that time.The Squamous Cell Carcinoma Antigen (SCC-Ag) level was 9.1ng/ml and was higher than the normal level. He underwent a needle biopsy of a left inguinal lymph node, with pathology confirming metastatic PSCC. Next-generation sequencing (NGS) testing showed PD-L1 protein expression with a Tumor Proportion Score (TPS) of 50% and a Combined Positive Score (CPS) of 60%. It was Microsatellite Stable (MSS). Tumor Mutation Burden(TMB) was 8 mutation/Mb. HPV was negative. It showed that PIK3CA exon 10 p.E542K missense mutation and KEAP1 exon 3 p.Asn382Ser c.1145A>G. The Copy Number Variation (CNV) Plot was showed in **Supplementary Figure S1**. A subsequent PET-CT scan was performed. It demonstrated post-surgical changes consistent with the prior penile cancer resection. No mass or increased FDG uptake was identified at the surgical bed or the penile stump. However, multiple enlarged lymph nodes were noted adjacent to the left external iliac vessels in the inguinal region, with the largest measuring approximately 2.0 × 3.1 cm. Some of these nodes appeared confluent and demonstrated increased FDG avidity, with a maximum standardized uptake value (SUVmax) of 7.2 and an average SUV (SUVavg) of 6.3 (**Figure 1**). Enhanced Magnetic Resonance Imagin (MRI) suggests metastasis in the left inguinal lymph nodes (**Supplementary Figure S2**). Following a multidisciplinary team (MDT) discussion at our hospital, the patient’s advanced age and inability to tolerate chemotherapy were taken into consideration. A comprehensive treatment plan of radiotherapy combined with immunotherapy was formulated. For radiotherapy planning, the patient was positioned supine and immobilized using a vacuum bag. A non-contrast plus contrast-enhanced CT simulation scan was performed. The gross tumor volume of the nodes (GTVn) was delineated based on the enlarged lymph nodes identified on the prior PET/CT. The gross tumor volume (GTVn) of the left inguinal metastatic lymph nodes was expanded uniformly by 5 mm in all directions to generate the clinical target volume (CTV). A 3-mm expansion from the CTV was used to create the planning target volume (PTV), limited to the external body contour. The prescribed doses were 60 Gy in 30 fractions to the PTV. The treatment plan was generated using helical tomography (TOMO), the dose distribution map, overlaid on the CTV, is illustrated in **Figure 2**. He was concurrent receiving of Pembrolizumab (200 mg q3w). After the course of radiation therapy, the lower extremity edema was resolved.The patient presented with grade 2 radiation dermatitis at the end of radiotherapy and was resolved by management of Bepanthen Ointment.Leukopenia (grade 1) and fatigue (garde 2) were resolved using symptomatic drug treatment. The patient continued regular Pembrolizumab immunotherapy with surveillance comprising imaging and SCC-Ag level checks. The toxicity was well tolerance, with no grade ≥ 3 adverse events.During the couse of immunotherapy, fatigue (garde 2), mild anemia(grade 1) and hypokalemia (grade 2) was resoveld by symptomatic treatment. Imaging examination followed-up was performed by CT, MRI or PET-CT according to patients choice.every 3 months. CT or MRI evaluation criteria was according to the iRECIST standards, PET-CT evaluation criteria was according to PERCIST. In February 2023, a Computed Tomography scan indicated the patient had a partial response disease. In January 2024, the MRI scan indicated that the patients had a nearly clinical complete response (cCR) (**Supplementary Figure S2**).Clinical complete response (cCR) was defined as the complete disappearance of all palpable inguinal lymph nodes on physical examination, combined with the absence of any residual or new fluorodeoxyglucose (FDG)-avid lesions on follow-up ^18^F-FDG PET/CT performed at 3 and 12 months post-treatment, corresponding to a Deauville score of 1–2. This definition aligns with the established PERCIST 1.0 criteria and consensus recommendations for response assessment in solid tumors. And the SCC-Ag level was back to normal level. He received 34 cycles of Pembrolizumab treatment the last time in July 2024. A PET-CT scan was performed in May 2025, follow-up imaging demonstrated no evidence of local tumor recurrence at the primary surgical site and showed no FDG-avid lymph nodes in the left inguinal region (**Figure 3**). No enlarged lymph nodes were palpable in the left inguinal region. SCC-Ag, blood routine, liver function, kidney function, electrolyte, thyroid function and physical examination were regularly measured. Furthermore, no disease progression was observed. The patient had a cCR after two years of treatment. The timeline scheme of major clinical event of the patients since diagnosis and the changes of SCC-Ag levels were showed in **Figure 4**.

## Discussion

In this case, the very advanced old patient with PSCC with multiple metastases to the left inguinal lymph nodes received radiotherapy and pembrolizumab treatment and achivied cCR It was with a high CPS of 60%.The progression-free survival (PFS) was > 38 months at the last calculation with no grade ≥ 3 adverse events. Radiotherapy plus immunotherapy for the management of PSCC have been reported in several studies.The differences between the present case and previous reports was this patient with a very advanced age, and treatment with concurrent schedule with TOMO technique.

The therapeutic strategy for the primary lesion in PSCC centers on achieving local cure without compromising penile structure and function (21). Treatment options for localized penile cancer are not supported by randomized or comparative trial data. A histologically confirmed diagnosis and local tumor stage are mandatory before any non-surgical intervention. The histology crucially defines the tumor’s risk group, which in turn dictates the approach to invasive staging of the groins (10).

The management of recurrent and metastatic PSCC has undergone a significant paradigm shift. The primary goal is no longer curative but focuses on prolonging survival, controlling symptoms, and preserving quality of life. Treatment selection is highly individualized, depending on the patient’s performance status, prior therapies, and tumor molecular profile, and mandates a multidisciplinary approach.

The treatment backbone for metastatic PSCC has evolved from chemotherapy alone to combination regimens incorporating immunotherapy.Platinum-based regimens remain a foundational option, particularly for patients ineligible for immunotherapy. While offering objective response rates (ORR) of approximately 50%, their utility is limited by significant toxicity and modest median OS of around 17 months (8). In this case, because of patients with older age and unwilling to received chemotherapy.

In the ORPHEUS trial (22), the clinical activity and safety of the PD-1 inhibitor retifanlimab were evaluated in a cohort of 18 treatment-naïve, advanced PSCC patients. The regimen (500 mg every 4 weeks) yielded an ORR of 16.7% and a disease control rate (DCR) of 22.2%, with a median duration of response of 3.3 months. Survival outcomes included a median PFS of 2.0 months and a median OS of 7.2 months. Treatment was characterized by a manageable safety profile, with a low incidence of grade 3 toxicities. Another real-world cohort comprised 92 patients with metastatic PSCC (90%), most (80%) of whom had received ≥2 prior lines of therapy. With a median follow-up not specified, the median OS and PFS were 9.8 months and 3.2 months, respectively. The ORR was 13% overall but reached 35% in the subgroup with lymph node-only metastases (23). These findings, though preliminary due to the small sample size, indicate that retifanlimab has antitumor activity in advanced PSCC and support its further investigation in this population.

In our case, the tumor showed PD-L1 protein expression with a TPS of 50%, indicating potential sensitivity to immunotherapy. This patients had a negattive of HPV, other studies also suggested that in HPV -PSCC, immutnotherapy is a potential treatment approach (15, 24).Used ICI to PSCC still need to more clinical trials to confirm.

Local therapies retain a crucial, albeit more selective, role in the metastatic setting for symptom control and addressing oligometastatic disease. Surgery was carefully considered. For isolated local recurrence, surgical excision (re-resection or salvage penectomy) remains the primary curative-intent modality. For regional nodal recurrence, therapeutic lymph node dissection can be offered with palliative or, in select cases, curative intent, always weighing benefits against morbidity and quality of life (25).Radiotherapy is a key tool for local control, adjuvant radiotherapy and palliation. Systematic evidence from a review of 14 studies provides support for adjuvant radiotherapy in high-risk penile cancer. In patients with high-risk features (R1 resection or extranodal extension), this approach translated into a superior median disease-specific survival of 14.4 months, a statistically significant improvement over the 8.0 months observed with surveillance (P = 0.02) (26). It is effectively used for unresectable local or regional recurrences and palliation of symptomatic metastases (e.g., bone, brain). Radiotherapy may also be combined synergistically with systemic therapies like immunotherapy (27–29).

In our case, the helical tomotherapy (TOMO) was applied. For patients with lesions near critical structures, helical tomotherapy (TOMO) offers clear dosimetric benefits, especially in target dose uniformity and organ-at-risk(OAR) protection (30). Using spiral irradiation, TOMO achieves highly conformal and homogeneous dose coverage in irregular targets, avoiding field-matching issues common with conventional techniques. Its dynamic multileaf collimator and helical delivery further refine dose shaping, minimize high-dose exposure to normal tissues, and reduce the risk of associated side effects (31).

In our case, the patients was in a very advanced age, data on immunotherapy in patients aged over 80 years remain limited.The benefit of immunotherapy in lung cancer is well-established: among patients aged ≥80 years with advanced Non-Small Cell Lung Cancer receiving first-line ICIs (predominantly as monotherapy), PFS is comparable to that in younger patients, and toxicity shows no linear correlation with age (32). Onother study also indiacated that the incidence of immune-related adverse events(iRAEs) and treatment discontinuation rates showed no statistically significant differences between older and younger patient groups (33, 34). Although combination therapy was associated with numerically higher toxicity compared to monotherapy, age was not identified as an independent risk factor. In our case, the treatment did not increse iRAEs.

Given the distinct biology of HPV-driven tumors, a treatment strategy combining radiotherapy and immunotherapy could offer greater clinical benefit over standard chemotherapy in PSCC (35). Investigating the combination of atezolizumab and radiotherapy in advanced penile cancer, the phase II PERICLES trial (19) (N = 32) yielded a 1-year PFS of 12.5% and a median OS of 11.3 months. The overall ORR was 16.7% (n=30 evaluable), with no differential effect observed between the combination and monotherapy arms. Notably, a biomarker analysis revealed that patients harboring high-risk HPV had a longer median PFS (5.3 months) than HPV-negative patients (2.6 months). Another report also indicated that pembrolizumab and stereotactic body radiation therapy in recurrent metastatic PSCC was effective and safety (36). Our case using radiotherapy concurrent immunotherapy, because this patient was metastatic PSCC, and did not received systematic chemotherapy, considering the systematic treatment was important, the concurrent strategy was adopted. Moreover, a superior outcome was observed, characterized by a progression-free survival of more than 38 months.

The two-year treatment duration for PD-1/PD-L1 inhibitors is based on pharmacological rationale, pivotal trial designs, long-term outcomes, and risk-benefit assessment.PD-1/PD-L1 inhibitors reinvigorate tumor-specific T-cells. A defined treatment period allows for adequate T-cell expansion and differentiation into long-lived memory T-cells, which may sustain anti-tumor immunity after discontinuation.The two-year schedule originates from key registration trials including KEYNOTE trials (37) and CheckMate trials (38), treatment until progression or toxicity, often capped at 2 years.Similar frameworks were used in melanoma, renal cell carcinoma, and head and neck cancer trials.This duration balanced efficacy induction with toxicity and cost concerns.Patients completing 2 years of therapy show sustained benefits and Low relapse after cCR(<10%).Moreover, late immune-related adverse events are limited, Improves cost-effectiveness and facilitates healthcare planning. In our case, the patient did not occur more than grade 3 treatment-related events and the treatment is tolerable.

The patient was with a CPS of 60%, Some studies indicated that High CPS Scores was associated with greater benefit, but thresholds Are Cancer-Specific. CPS ≥1 remains the essential enrollment threshold for PD-1/PD-L1 inhibitors across most indications. Higher CPS cutoffs (≥20, ≥50) inform treatment optimization (monotherapy vs. combination) but do not define absolute eligibility.CPS is a density metric with intrinsic limitations: it disregards spatial architecture, immune cell functionality, and host systemic factors (39–41). This pateitn with a higher CPS was benifits with immunotherapy.

While PD-L1 expression alone has variable predictive value, a tumor microenvironment rich in CD8+ T-cell infiltration is strongly associated with superior outcomes from PD-1 blockade, especially in combination regimens (10, 15). Tumors with dMMR/MSI-H or high TMB are candidates for PD-1 inhibitor monotherapy (42, 43).However, our case was MSS.Given the rarity of PSCC, enrollment in clinical trials is strongly encouraged. Active research areas include novel immunotherapy combinations (e.g., dual checkpoint inhibition, immunotherapy with targeted agents) and therapies specifically targeting HPV-related pathways.

A key limitation in this case is the absence of a post-treatment biopsy of the left inguinal lymph nodes. Consequently, a pCR could not be confirmed.

## Conclusion

The management of recurrent and metastatic PSCC should be integrated multimodal strategies. This case suggests that concurrent radiotherapy plus Pembrolizumab may achieve durable remission in elderly, chemotherapy-intolerant, metastatic PSCC, but verification in more patients is needed.

## Data Availability

The original contributions presented in the study are included in the article/**Supplementary Material**. Further inquiries can be directed to the corresponding author.

## Supplementary material

Supplementary Figure 1 Copy Number Variation (CNV) Plot. It illustrates the copy number distribution across all genes. Each point represents a capture interval for a gene, with color-highlighted points indicating genes of interest harboring copy number variations. The x-axis denotes the chromosomal position of the genes, and the y-axis represents the copy number calculated based on the NGS methodology (the red horizontal line indicates the normal gene copy number). It should be noted that the copy number derived from NGS analysis may be diluted by normal cell DNA present in the tested sample and therefore may not represent the absolute copy number per cell.

Supplementary Figure 2 Baseline and 15-month followed-up MRI.The baseline MRI revealed metastatic involvement of the left inguinal lymph node., 15-month followed-up imaging revealed that lymph nodes in the left inguinal region achieved clinical complete response.

## References

[1] Hernandez BY Barnholtz-Sloan J German RR Giuliano A Goodman MT King JB . Burden of invasive squamous cell carcinoma of the penis in the United States, 1998-2003. Cancer. (2008) 113:2883–91. doi: 10.1002/cncr.23743, PMID: 18980292 PMC2693711

[2] Wang Y Wang K Chen Y Zhou J Liang Y Yang X . Mutational landscape of penile squamous cell carcinoma in a Chinese population. Int J Cancer. (2019) 145:1280–89. doi: 10.1002/ijc.32373, PMID: 31034097

[3] Fernández-Nestosa MJ Sanchez DF Cañete-Portillo S Alemany L Clavero O Lloveras B . Human papillomavirus (HPV) genotypes in mixed squamous cell carcinoma of the penis: A study of 101 tumors. Int J Surg Pathol. (2025) 33:877–81. doi: 10.1177/10668969241295352, PMID: 39535004

[4] Olesen TB Sand FL Rasmussen CL Albieri V Toft BG Norrild B . Prevalence of human papillomavirus DNA and p16(INK4a) in penile cancer and penile intraepithelial neoplasia: a systematic review and meta-analysis. Lancet Oncol. (2019) 20:145–58. doi: 10.1016/S1470-2045(18)30682-X, PMID: 30573285

[5] Pekarek L Ortega MA Fraile-Martinez O García-Montero C Casanova C Saez MA . Clinical and novel biomarkers in penile carcinoma: A prospective review. J Pers Med. (2022) 12:1364. doi: 10.3390/jpm12091364, PMID: 36143149 PMC9502223

[6] Johnston MJ Nigam R . Recent advances in the management of penile cancer. F1000Research. (2019) 8:F1000–558. doi: 10.12688/f1000research.18185.1, PMID: 31069061 PMC6490003

[7] Leijte JAP Kirrander P Antonini N Windahl T Horenblas S . Recurrence patterns of squamous cell carcinoma of the penis: recommendations for follow-up based on a two-centre analysis of 700 patients. Eur Urol. (2008) 54:161–68. doi: 10.1016/j.eururo.2008.04.016, PMID: 18440124

[8] Di Lorenzo G Federico P Buonerba C Longo N Cartenì G Autorino R . Paclitaxel in pretreated metastatic penile cancer: final results of a phase 2 study. Eur Urol. (2011) 60:1280–84. doi: 10.1016/j.eururo.2011.08.028, PMID: 21871710

[9] Zhang Q Li Y Zhang Y Deng Z Ding Y . Case report of penile squamous cell carcinoma continuous treatment with BRCA2 mutation. World J Surg Oncol. (2024) 22:50. doi: 10.1186/s12957-024-03305-9, PMID: 38336701 PMC10854037

[10] Thomas A Necchi A Muneer A Tobias-MaChado M Tran ATH Van Rompuy A . Penile cancer. Nat Rev Dis Primers. (2021) 7:11. doi: 10.1038/s41572-021-00246-5, PMID: 33574340

[11] McGregor BA Campbell MT Xie W Farah S Bilen MA Schmidt AL . Results of a multicenter, phase 2 study of nivolumab and ipilimumab for patients with advanced rare genitourinary Malignancies. Cancer. (2021) 127:840–49. doi: 10.1002/cncr.33328, PMID: 33216356 PMC13213840

[12] Simon N Atiq S Sonpavde G Apolo A . New therapeutic horizons for advanced or metastatic penile cancer. Urol Clinics North America. (2024) 51:367–76. doi: 10.1016/j.ucl.2024.03.005, PMID: 38925739 PMC11290867

[13] Tang Y Hu X Wu K Li X . Immune landscape and immunotherapy for penile cancer. Front Immunol. (2022) 13:1055235. doi: 10.3389/fimmu.2022.1055235, PMID: 36524123 PMC9745054

[14] Maisch P Koll F Bolenz C Chun FK Gschwend JE Schmid SC . Combination of radiation and immunotherapy in the treatment of genitourinary Malignancies: A systematic review and meta-analysis. Urol Oncol. (2023) 41:219–32. doi: 10.1016/j.urolonc.2022.10.009, PMID: 36372634

[15] Taghizadeh H Fajkovic H . Immunotherapy in the management of penile cancer-A systematic review. Cancers (Basel). (2025) 17:883. doi: 10.3390/cancers17050883, PMID: 40075730 PMC11898862

[16] Galluzzi L Yamazaki T Kroemer G . Linking cellular stress responses to systemic homeostasis. Nat Rev Mol Cell Biol. (2018) 19:731–45. doi: 10.1038/s41580-018-0068-0, PMID: 30305710

[17] Liao Y Deng J Yang X Wang D Du X . Advances in radiotherapy enhancing the efficacy of immune checkpoint inhibitors in Malignant. Front Oncol. (2025) 15:1611036. doi: 10.3389/fonc.2025.1611036, PMID: 40666094 PMC12259436

[18] Chen X Yang M Huang Y Tu J Cai Y Yuan X . Molecular mechanisms underlying the abscopal effect induced by radiotherapy and its synergistic translational potential with immunotherapy. Ther Adv Med Oncol. (2025) 17:17588359251387534. doi: 10.1177/17588359251387534, PMID: 41179116 PMC12579151

[19] de Vries HM Rafael TS Gil-Jimenez A de Feijter JM Bekers E van der Laan E . Atezolizumab with or without radiotherapy for advanced squamous cell carcinoma of the penis (The PERICLES study): A phase II trial. J Clin Oncol Off J Am Soc Clin Oncol. (2023) 41:4872–80. doi: 10.1200/JCO.22.02894, PMID: 37487169

[20] Riley DS Barber MS Kienle GS Aronson JK von Schoen-Angerer T Tugwell P . CARE guidelines for case reports: explanation and elaboration document. J Clin Epidemiol. (2017) 89:218–35. doi: 10.1016/j.jclinepi.2017.04.026, PMID: 28529185

[21] Garaz R Mirvald C Spiess PE Daniel Grass G Thomas A Surcel C . Brachytherapy and external beam radiation in the management of primary penile cancer - Game changer for organ preservation? Cancer Treat Rev. (2024) 129:102800. doi: 10.1016/j.ctrv.2024.102800, PMID: 39002212

[22] García Del Muro X Páez López-Bravo D Cuéllar-Rivas MA Maroto P Giannatempo P Castellano D . Retifanlimab in advanced penile squamous cell carcinoma: the phase 2 ORPHEUS study. Eur Urol Oncol. (2025) 8:278–86. doi: 10.1016/j.euo.2024.04.021, PMID: 38749903

[23] El Zarif T Nassar AH Pond GR Zhuang TZ Master V Nazha B . Safety and efficacy of immune checkpoint inhibitors in advanced penile cancer: report from the Global Society of Rare Genitourinary Tumors. J Natl Cancer Institute. (2023) 115:1605–15. doi: 10.1093/jnci/djad155, PMID: 37563779 PMC11032703

[24] Song H Tong Z Xie G Li Y Zhao Y Fan F . Single-cell and spatial transcriptomic profiling of penile squamous cell carcinoma reveals dynamics of tumor differentiation and immune microenvironment. Adv Sci (Weinh). (2025) 12:e00216. doi: 10.1002/advs.202500216, PMID: 40470730 PMC12412502

[25] Neuville P Escoffier A Savoie P Fléchon A Branger N Rocher L . French AFU cancer committee guidelines-update 2024-2026: penile cancer. French J Urol. (2024) 34:102736. doi: 10.1016/j.fjurol.2024.102736, PMID: 39581662

[26] Loos G Escande A Aziez S Marchesi V Sargos P Terzilli M . Radiotherapy for penile cancers: 2025 update. Cancer Radiother: J La Societe Francaise Radiother Oncol. (2025) 29:104761. doi: 10.1016/j.canrad.2025.104761, PMID: 41205450

[27] Leone A Diorio GJ Pettaway C Master V Spiess PE . Contemporary management of patients with penile cancer and lymph node metastasis. Nat Rev Urol. (2017) 14:335–47. doi: 10.1038/nrurol.2017.47, PMID: 28401957

[28] Jaime-Casas S Barragan-Carrillo R Eskenazi F Dugarte JP Chahoud J Spiess PE . Evaluating the evolving treatment landscape of systemic therapies in penile cancer. Cancers (Basel). (2025) 17:2956. doi: 10.3390/cancers17182956, PMID: 41008800 PMC12468617

[29] Korzeniowski MA Crook JM . Contemporary role of radiotherapy in the management of penile cancer. Transl Androl Urol. (2017) 6:855–67. doi: 10.21037/tau.2017.07.02, PMID: 29184783 PMC5673811

[30] Sterzing F Engenhart-Cabillic R Flentje M Debus J . Image-guided radiotherapy: a new dimension in radiation oncology. Dtsch Arztebl Int. (2011) 108:274–80. doi: 10.3238/arztebl.2011.0274, PMID: 21603562 PMC3097488

[31] Zhang Y Rong L Wang Z Zhao H . The top 100 most cited articles in helical tomotherapy: a scoping review. Front Oncol. (2023) 13:1274290. doi: 10.3389/fonc.2023.1274290, PMID: 37916164 PMC10616822

[32] Del Corral-Morales J Ayala-de Miguel C Quintana-Cortes L Sanchez-Vegas A Aranda-Bellido F Gonzalez-Santiago S . Real-world data on immune-checkpoint inhibitors in elderly patients with advanced non-small cell lung cancer: A retrospective study. Cancers (Basel). (2025) 17:2194. doi: 10.3390/cancers17132194, PMID: 40647491 PMC12248468

[33] Taylor JS White C Collins L Moran B Kukard C . Effectiveness and toxicity of anticancer agents in older Australian patients: A retrospective analysis. Asia Pac J Clin Oncol. (2025) 21:616–22. doi: 10.1111/ajco.14186, PMID: 40348594 PMC12582344

[34] Yao J Li S Bai L Chen J Ren C Liu T . Efficacy and safety of immune checkpoint inhibitors in elderly patients with advanced non-small cell lung cancer: a systematic review and meta-analysis. EClinicalMedicine. (2025) 81:103081. doi: 10.1016/j.eclinm.2025.103081, PMID: 39975700 PMC11836518

[35] Longoni M Fankhauser CD Negri F Salonia A Basile G Johnstone PAS . Treatment strategies in human papillomavirus-related advanced penile cancer. Nat Rev Urol. (2025) 22:427–38. doi: 10.1038/s41585-025-00994-z, PMID: 39966660

[36] Kaakour D Seyedin S Houshyar R Mar N . Combination of pembrolizumab and stereotactic body radiation therapy in recurrent metastatic penile squamous cell carcinoma: A case study. Biomedicines. (2022) 10:3033. doi: 10.3390/biomedicines10123033, PMID: 36551787 PMC9775235

[37] Garon EB Rizvi NA Hui R Leighl N Balmanoukian AS Eder JP . Pembrolizumab for the treatment of non-small-cell lung cancer. N Engl J Med. (2015) 372:2018–28. doi: 10.1056/NEJMoa1501824, PMID: 25891174

[38] Hodi FS Chiarion-Sileni V Gonzalez R Grob J Rutkowski P Cowey CL . Nivolumab plus ipilimumab or nivolumab alone versus ipilimumab alone in advanced melanoma (CheckMate 067): 4-year outcomes of a multicentre, randomised, phase 3 trial. Lancet Oncol. (2018) 19:1480–92. doi: 10.1016/S1470-2045(18)30700-9, PMID: 30361170

[39] Tashireva LA Kalinchuk AY Shmakova EO Tsarenkova EA Loos DM Iamschikov P . PD-1-positive CD8+ T cells and PD-1-positive foxP3+ Cells in tumor microenvironment predict response to neoadjuvant chemoimmunotherapy in gastric cancer patients. Cancers (Basel). (2025) 17:2407. doi: 10.3390/cancers17142407, PMID: 40723289 PMC12293087

[40] Ohara A Mori T Itoyama M Yokoyama K Yamamoto S Kato K . Relationship between short-term outcomes and PD-L1 expression based on combined positive score and tumor proportion score in recurrent or metastatic head and neck cancers treated with anti-PD-1 antibody monotherapy. Cancer Rep (Hoboken). (2025) 8:e70125. doi: 10.1002/cnr2.70125, PMID: 39840665 PMC11751707

[41] Kuang X Xu R Li J . Association of PD-L1 expression with survival benefit from PD-1/PD-L1 inhibitors in advanced cancer: Systematic review and meta-analysis of phase III randomized clinical trials. Crit Rev Oncol Hematol. (2024) 198:104357. doi: 10.1016/j.critrevonc.2024.104357, PMID: 38614270

[42] Nazha B Zhuang T Wu S Brown JT Magee D Carthon BC . Comprehensive genomic profiling of penile squamous cell carcinoma and the impact of human papillomavirus status on immune-checkpoint inhibitor-related biomarkers. Cancer. (2023) 129:3884–93. doi: 10.1002/cncr.34982, PMID: 37565840

[43] Tanegashima T Shiota M Toyosaki K Funakoshi K Eto M . Biology and evolving management of resectable dMMR/MSI-H cancers: current status and future perspectives. Cancer Immunol Immunother: CII. (2025) 74:364. doi: 10.1007/s00262-025-04223-9, PMID: 41196394 PMC12592604

