# Supplementary figures and images for: Radiotherapy combined with immunotherapy achieves clinical complete response in elderly metastatic penile cancer: a case report
Source: Front Immunol. 2026 Mar 9;17:1783437. doi: 10.3389/fimmu.2026.1783437 (PMC13006661; doi:10.3389/fimmu.2026.1783437)

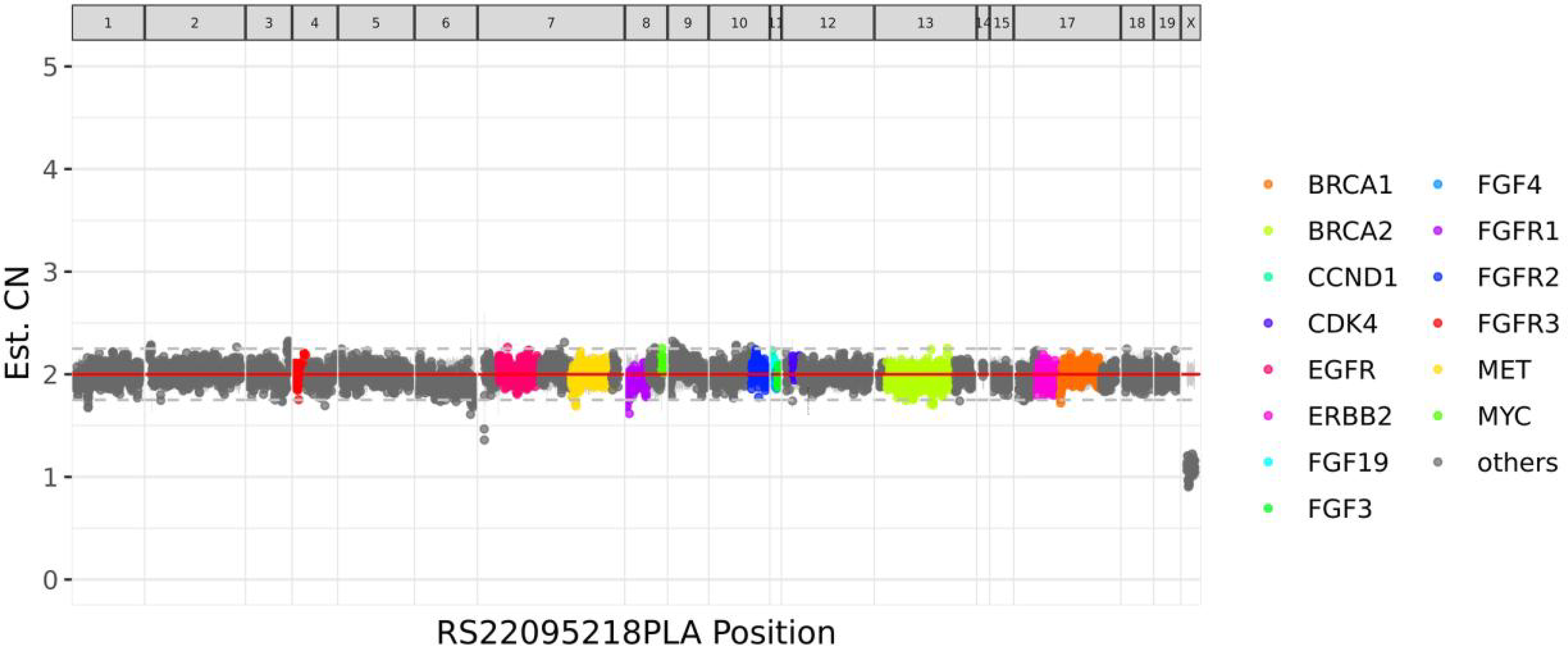

Supplement: Supplementary Figure 1 — Copy Number Variation (CNV) Plot. It illustrates the copy number distribution across all genes. Each point represents a capture interval for a gene, with color-highlighted points indicating genes of interest harboring copy number variations. The x-axis denotes the chromosomal position of the genes, and the y-axis represents the copy number calculated based on the NGS methodology (the red horizontal line indicates the normal gene copy number). It should be noted that the copy number derived from NGS analysis may be diluted by normal cell DNA present in the tested sample and therefore may not represent the absolute copy number per cell. [file Image1.tif]

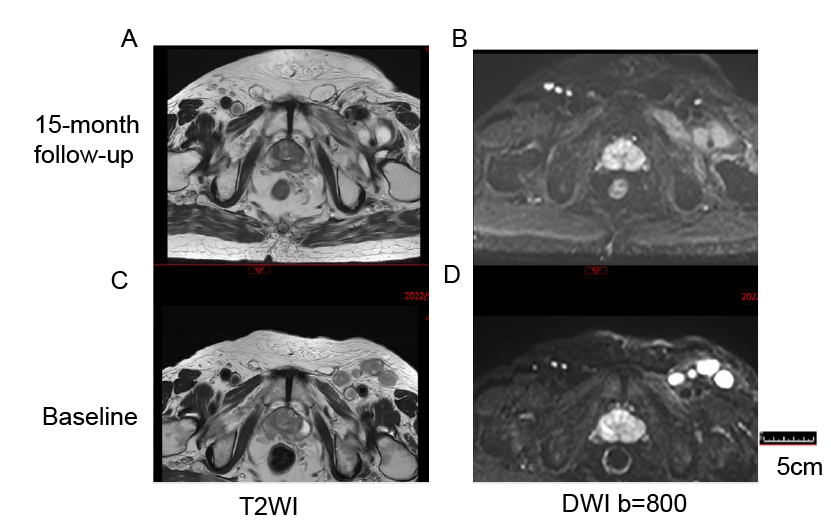

Supplement: Supplementary Figure 2 — Baseline and 15-month followed-up MRI.The baseline MRI revealed metastatic involvement of the left inguinal lymph node., 15-month followed-up imaging revealed that lymph nodes in the left inguinal region achieved clinical complete response. [file Image2.tif]
